# Supplementary material for: FgAP1σ Is Critical for Vegetative Growth, Conidiation, Virulence, and DON Biosynthesis in Fusarium graminearum
Source: J Fungi (Basel). 2023 Jan 21;9(2):145. doi: 10.3390/jof9020145 (PMC9962196; doi:10.3390/jof9020145)
Supplement: Supplementary file 1 [file jof-09-00145-s001.zip › jof-2165770-supplementary.pdf]

FgAP1$^{\sigma}$ Is Critical for Vegetative Growth, Conidiation, Virulence, and DON Biosynthesis in *Fusarium graminearum*

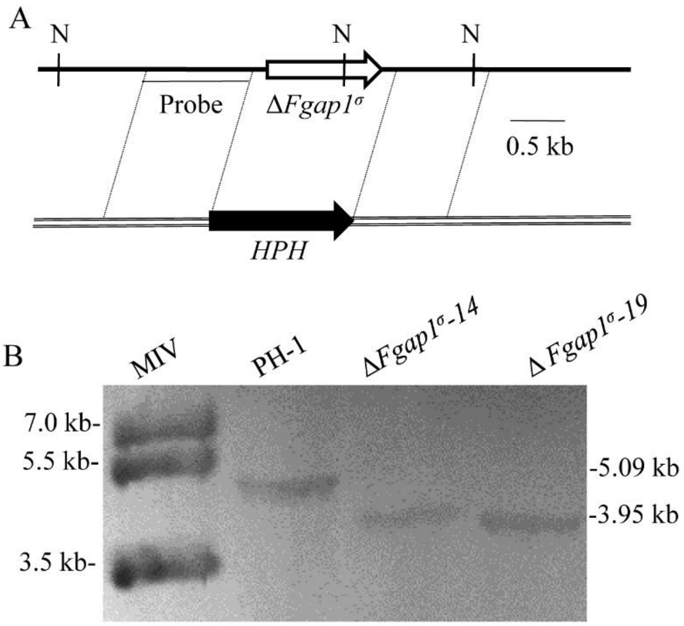

Figure S1. Southern blot analysis of the *FgAP1$^{\sigma}$* deletion mutants. Genomic DNA was extracted from PH-1 and the positive transformants and digested with restriction enzymes. (A) Deletion strategy for *FgAP1$^{\sigma}$* and its Southern blot confirmation. (B) *Nco*I DNA digestion showed a 5.09 kb band in the wild-type PH-1 and a 3.95 kb band in the Δ*Fgap1$^{\sigma}$* mutants.

Table S1. Fungal strains used in this study.

Strain	Genotype description	Reference
PH-1	Wild-type	(Cuomo et al., 2007)
Δ*Fgap1$^{\sigma}$-14*	FGSG_10034 deletion mutant from PH-1	This study
Δ*Fgap1$^{\sigma}$-19*	FGSG_10034 deletion mutant from PH-1	This study
Δ*Fgap1$^{\sigma}$-C*	Δ*Fgap1$^{\sigma}$* strain expressing FgAP1$^{\sigma}$-GFP construct	This study
PH-1+ FgAP1$^{\sigma}$-GFP + FgKex2-mCherry	PH-1 strain expressing FgAP1$^{\sigma}$-GFP and FgKex2-mCherry constructs	This study

PH-1+ FgAP1$^{\beta}$*-GFP + FgKex2-mCherry*	PH-1 strain expressing FgAP1$^{\beta}$-GFP and FgKex2-mCherry constructs	This study
PH-1+ FgAP1$^{\gamma}$*-GFP + FgKex2-mCherry*	PH-1 strain expressing FgAP1$^{\gamma}$-GFP and FgKex2-mCherry constructs	This study
PH-1+ FgAP1$^{\mu}$*-GFP + FgKex2-mCherry*	PH-1 strain expressing FgAP1$^{\mu}$-GFP and FgKex2-mCherry constructs	This study
Δ*Fgap1*$^{\sigma}$*+FgAP1*$^{\beta}$*-GFP*	Δ*Fgap1*$^{\sigma}$ strain expressing *FgAP1*$^{\beta}$*-GFP* construct	This study
Δ*Fgap1*$^{\sigma}$*+FgAP1*$^{\gamma}$*-GFP*	Δ*Fgap1*$^{\sigma}$ strain expressing *FgAP1*$^{\gamma}$*-GFP* construct	This study
Δ*Fgap1*$^{\sigma}$*+FgAP1*$^{\mu}$*-GFP*	Δ*Fgap1*$^{\sigma}$ strain expressing *FgAP1*$^{\mu}$*-GFP* construct	This study
PH-1+GFP-FgSnc1	PH-1 strain expressing *GFP-FgSnc1*construct	This study
Δ*Fgap1*$^{\sigma}$*+GFP-FgSnc1*	Δ*Fgap1*$^{\sigma}$ strain expressing *GFP-FgSnc1* construct	This study

Cuomo, C.A., Guldener, U., Xu, J.R., Trail, F., Turgeon, B.G., Di Pietro, A. et al. (2007) The Fusarium graminearum genome reveals a link between localized polymorphism and pathogen specialization. *Science*, 317,1400-1402.

Table S2. Primers used in this study.

Primers	Sequence 5'-3'	Annotation
FgAP1$^{\sigma}$-AF	GCCGAGTATGTGCCCTCTTG	*FgAP1*$^{\sigma}$ deletion and Southern probe
FgAP1$^{\sigma}$-AR	TTGACCTCCACTAGCTCCAGCCAAGCCCTTGCCTGGATCGACAACTCA	
FgAP1$^{\sigma}$-BF	GAATAGAGTAGATGCCGACCGCGGGTTGGAGACGGGAGTTCTGTAATGTG	*FgAP1*$^{\sigma}$ deletion
FgAP1$^{\sigma}$-BR	CGTGGCATCCGTGGTAATAGC	
FgAP1$^{\sigma}$-OF	TCTCCCGCCTGCTAATCAAGAC	*FgAP1*$^{\sigma}$ deletion
FgAP1$^{\sigma}$-OR	ATGATCTTTGTAACTTCGTCCTC	
FgAP1$^{\sigma}$-UA	CTAACATCTCGCAAGGCTCCTC	*FgAP1*$^{\sigma}$ deletion
FgAP1$^{\gamma}$-AF	AATGACACCCTCGGACAAGC	*FgAP1*$^{\gamma}$ deletion
FgAP1$^{\gamma}$-AR	TTGACCTCCACTAGCTCCAGCCAAGCCAGTTTGGCAGGAGCCTATCC	
FgAP1$^{\gamma}$-BF	GAATAGAGTAGATGCCGACCGCGGGTTG	*FgAP1*$^{\gamma}$ deletion

	GGGTGGACTTGAAGAAGATAGA	
FgAP1$^{\gamma}$-BR	CCTACACCTCCCGCTATGAAC	
FgAP1$^{\gamma}$-OF	ATGTTACTGGCATTACCGACCCT	*FgAP1$^{\gamma}$* deletion
FgAP1$^{\gamma}$-OR	AGATGTCAGCGAGAAGGTCAGT	
FgAP1$^{\gamma}$-UA	CCAGTCCCAATCTGCCTCAC	*FgAP1$^{\gamma}$* deletion
FgAP1$^{\beta}$-AF	AGAGGCGAGCACAAACGAAT	*FgAP1$^{\beta}$* deletion
FgAP1$^{\beta}$-AR	TTGACCTCCACTAGCTCCAGCCAAGCCC	
	GTATCGGGAGATGCGTCAA	
FgAP1$^{\beta}$-BF	GAATAGAGTAGATGCCGACCGCGGGTTAA	*FgAP1$^{\beta}$* deletion
	CCATCTTGTTATCGCGTCAAA	
FgAP1$^{\beta}$-BR	ATGCGTTGGATAGTCTGGGATAA	
FgAP1$^{\beta}$-OF	TGACCGCTCTGTCTGAGATTACC	*FgAP1$^{\beta}$* deletion
FgAP1$^{\beta}$-OR	GTCTGAAGAAAGAAGACGCCAGTA	
FgAP1$^{\beta}$-UA	AAGGCCAACCGGCAGTTCTAT	*FgAP1$^{\beta}$* deletion
FgAP1$^{\mu}$-AF	CGTGGTTGGCTGTTGTGAAGAC	*FgAP1$^{\mu}$* deletion
FgAP1$^{\mu}$-AR	TTGACCTCCACTAGCTCCAGCCAAGCCG	
	CAAGATTGTAATGGGTTGGTTTA	
FgAP1$^{\mu}$-BF	GAATAGAGTAGATGCCGACCGCGGGTTC	*FgAP1$^{\mu}$* deletion
	GAACAACAGCGTTCTCCCAC	
FgAP1$^{\mu}$-BR	GGGTGTAGGACTGACCGAGAAT	
FgAP1$^{\mu}$-OF	CGAAATCCTATTGTTCCTCCAC	*FgAP1$^{\mu}$* deletion
FgAP1$^{\mu}$-OR	TTGCCTACTCCTCCAACACCTC	
FgAP1$^{\mu}$-UA	TCCTGATGCGTTTGAAGGTTGT	*FgAP1$^{\mu}$* deletion
HYG/F	GGCTTGGCTGGAGCTAGTGGAGGTCAA	deletion
HY/R	GTATTGACCGATTCCTTGCGGTCCGAA	deletion
YG/F	GATGTAGGAGGGCGTGGATATGTCCT	deletion
HYG/R	AACCCGCGGTCGGCATCTACTCTATTC	deletion
H853	GACAGACGTCGCGGTGAGTT	deletion
FgAP1$^{\sigma}$-CF	AGGGAACAAAAGCTGGGTACCTCTGCCC	FgAP1$^{\sigma}$-GFP vector

	AATCAACTTCTG	
FgAP1$^{\sigma}$-CR	GCCGCCGCCGCCGCCAAGCTTCATGATCTTTGTAACTTCGTCC	
FgAP1$^{\gamma}$-CF	AGGGAACAAAAGCTGGGTACCCGTCCAGGATGTCGTGAA	FgAP1$^{\gamma}$-GFP vector
FgAP1$^{\gamma}$-CR	GCCGCCGCCGCCGCCAAGCTTAGACTCTGTCCAGTTGACTTGAT	
FgAP1$^{\beta}$-CF	AGGGAACAAAAGCTGGGTACCTGGCTCAAGGTCAAGAAAG	FgAP1$^{\beta}$-GFP vector
FgAP1$^{\beta}$-CR	GCCGCCGCCGCCGCCAAGCTTCAACAGACCCAGCAAGTCG	
FgAP1$^{\mu}$-CF	AGGGAACAAAAGCTGGGTACCGCTCATCTTTGAGTGCCTCG	FgAP1$^{\mu}$-GFP vector
FgAP1$^{\mu}$-CR	GCCGCCGCCGCCGCCAAGCTTGACTGCGTCGGGAAGTCGT	
FgAP1$^{\beta}$-BDF	CTGATCTCAGAGGAGGACCTGCATATGGCAGTAAATCGCATTA	FgAP1$^{\beta}$-BD vector
FgAP1$^{\beta}$-BDR	CGCTGCAGGTCGACGGATCCCCGGGAACTACAACAGACCCAGCAAGT	
FgAP1$^{\gamma}$-ADF	GACGTACCAGATTACGCTCATATGAGTTCCCTTAAGCAA	FgAP1$^{\gamma}$-AD vector
FgAP1$^{\gamma}$-ADR	TATCGATGCCCACCCGGGTGGAACTAAGACTCTGTCCAGTTGA	
FgAP1$^{\sigma}$-BDF	CTGATCTCAGAGGAGGACCTGCATATGGCGATCCACTACCTTA	FgAP1$^{\sigma}$-BD vector
FgAP1$^{\sigma}$-BDR	CGCTGCAGGTCGACGGATCCCCGGGAATTACATGATCTTTGTAACTTCGTCC	
FgAP1$^{\beta}$-ADF	GACGTACCAGATTACGCTCATATGGCAGTAAATCGCATT	FgAP1$^{\beta}$-AD vector

FgAP1$^{\beta}$-ADR	TATCGATGCCCACCCGGGTGGAACTACAACAGACCCAGCAAG	
FgAP1$^{\gamma}$-BDF	CTGATCTCAGAGGAGGACCTGCATATGAGTTCCCTTAAGCA	FgAP1$^{\gamma}$-BD vector
FgAP1$^{\gamma}$-BDR	CGCTGCAGGTCGACGGATCCCCGGGAACTAAGACTCTGTCCAGTTG	
FgAP1$^{\mu}$-ADF	GACGTACCAGATTACGCTCATATGGCTTCCGCACTATTCTT	FgAP1$^{\mu}$-AD vector
FgAP1$^{\mu}$-ADR	TATCGATGCCCACCCGGGTGGAATCAGACTGCGTCGGGAAGTC	
FgAP1$^{\mu}$-BDF	CTGATCTCAGAGGAGGACCTGCATATGGCTTCCGCACTATTCTTC	FgAP1$^{\mu}$-BD vector
FgAP1$^{\mu}$-BDR	CGCTGCAGGTCGACGGATCCCCGGGAATCAGACTGCGTCGGGAAGT	
GFPF	ATGGTGAGCAAGGGCGAGG	GFP
GFPR-TAA	CGACCTGCAGGCATGCAAGCTTTTACTTGTACAGCTCGTCCATGC	
mCherryF	ATGGTGAGCAAGGGCGAGG	mCherry
mCherryR-TAA	CGACCTGCAGGCATGCAAGCTTTTACTTGTACAGCTCGTCCATGC	
FgKex2PF	GGGCTTCAAGGGTTTCTACTC	TGN Golgi Marker
FgKex2OR	CTCGCCCTTGCTCACCATACGTCTACCGCCCAAAGG	
FgTRI1QF	TCCAGACTACGAAGTGCTA	qRT-PCR
FgTRI1QR	TCATCCTGTACCAATTCCAAT	
FgTRI4QF	ACCAGGTCCTCAGTCTTG	qRT-PCR
FgTRI4QR	TCGTTGTGCTTGCCATAG	
FgTRI5QF	TGAGGGATGTTGGATTGAGCAGTAC	qRT-PCR
FgTRI5QR	TGCTTCCGCTCATCAAACAGGT	

FgTRI6QF	GCTACTCAGAATGCCCTCAG	qRT-PCR
FgTRI6QR	CGCATGTTATCCACCCTGCTA	
FgTRI10QF	ATGATGATTGAGGATATGTTGT	qRT-PCR
FgTRI10QR	GAGTGATGAGGTCTGGAA	
FgTRI12QF	GCTGTAACTGTCCCCAGCAT	qRT-PCR
FgTRI12QR	GTGAAGTTGCGACCGTACTC	
Tubulin F	GTCAGTGCGGTAACCAAATCGGT	qRT-PCR
Tubulin R	CTCAGAGGTGCCGTTGTAAACACC	
